# Machine learning models for predicting of PD-1 treatment efficacy in Pan-cancer patients based on routine hematologic and biochemical parameters

**DOI:** 10.1186/s12935-024-03439-6

**Published:** 2024-07-21

**Authors:** Wenjian Yang, Cui Chen, Qiangqiang Ouyang, Runkun Han, Peng Sun, Hao Chen

**Affiliations:** 1 Department of Clinical Laboratory, State Key Laboratory of Oncology in South China, Guangdong Provincial Clinical Research Center for Cancer, Collaborative Innovation Center for Cancer Medicine, Guangdong Key Laboratory of Nasopharyngeal Carcinoma Diagnosis and Therapy, Sun Yat-Sen University Cancer Center, Guangzhou, 510060 People’s Republic of China; 2 Hangzhou Institute for Advanced Study, University of Chinese Academy of Sciences, Hangzhou, 310024 China; 3 Department of Oncology, The First Affiliated Hospital, Sun Yat-Sen University, 58 Zhongshan Road II, Guangzhou, 510080 China; 4 College of Electronic Engineering, South China Agricultural University, Guangzhou, 510642 Guangdong China; 5 Department of Medical Oncology, State Key Laboratory of Oncology in South China, Guangdong Provincial Clinical Research Center for Cancer, Collaborative Innovation Center for Cancer Medicine, Guangdong Key Laboratory of Nasopharyngeal Carcinoma Diagnosis and Therapy, Sun Yat-Sen University Cancer Center, Guangzhou, 510060 People’s Republic of China

**Keywords:** Pan-cancer, PD-1 checkpoint inhibitor, Machine learning, Hematologic, Biochemical

## Abstract

Immune checkpoint blockade therapy targeting the programmed death-1(PD-1) pathway has shown remarkable efficacy and durable response in patients with various cancer types. Early prediction of therapeutic efficacy is important for optimizing treatment plans and avoiding potential side effects. In this work, we developed an efficient machine learning prediction method using routine hematologic and biochemical parameters to predict the efficacy of PD-1 combination treatment in Pan-Cancer patients. A total of 431 patients with nasopharyngeal carcinoma, esophageal cancer and lung cancer who underwent PD-1 checkpoint inhibitor combination therapy were included in this study. Patients were divided into two groups: progressive disease (PD) and disease control (DC) groups. Hematologic and biochemical parameters were collected before and at the third week of PD-1 therapy. Six machine learning models were developed and trained to predict the efficacy of PD-1 combination therapy at 8–12 weeks. Analysis of 57 blood biomarkers before and after three weeks of PD-1 combination therapy through statistical analysis, heatmaps, and principal component analysis did not accurately predict treatment outcome. However, with machine learning models, both the AdaBoost classifier and GBDT demonstrated high levels of prediction efficiency, with clinically acceptable AUC values exceeding 0.7. The AdaBoost classifier exhibited the highest performance among the 6 machine learning models, with a sensitivity of 0.85 and a specificity of 0.79. Our study demonstrated the potential of machine learning to predict the efficacy of PD-1 combination therapy based on changes in hematologic and biochemical parameters.

**Supplementary Information:**

The online version contains supplementary material available at 10.1186/s12935-024-03439-6.

## Introduction

Immune checkpoint blockade (ICB) therapy targeting the programmed death-1 (PD-1) pathway has demonstrated remarkable efficacy and durable response in patients with various malignancies, including lung cancer (LC), melanoma, head and neck squamous cell carcinoma, esophageal carcinoma, and nasopharyngeal carcinoma (NPC) [1]. Although PD-1 antibodies have led to revolutionary advances in cancer therapy, their therapeutic efficacy varies greatly among individuals with the same cancer type. Therefore, it is of great clinical value to precisely target the beneficiary population of ICIs. Prior studies have demonstrated that biomarkers such as PD-L1 immunohistochemical expression, tumor mutation burden (TMB), tumor infiltrating lymphocytes (TILs), and instability/defective mismatch repair (MSI/dMMR) have the potential to predict the efficacy of immunotherapy. However, the current reliance on invasive biopsies for their assessment presents limitations in terms of accuracy. Consequently, there is a critical need to explore alternative noninvasive approaches to enhance the precision of predicting immunotherapy outcomes [2, 3].

Routine clinical laboratory tests, such as complete blood count (CBC) and routine biochemical analysis, which are prescribed for every patient receiving PD-1 antibodies, could serve as predictive biomarkers of PD-1 antibodies in previous studies [4]. In patients with multiple solid cancers, serum inflammatory indices, such as C-reactive protein (CRP) levels, lactate dehydrogenase (LDH) levels and the neutrophil-to-lymphocyte ratio (NLR), are traditionally recognized as hallmarks of the systemic immune-inflammatory status of the host [5]. In non-small cell lung cancer (NSCLC), the baseline derived-NLR (dNLR) and LDH level were combined to generate a novel prognostic model for patients treated with PD-1 antibodies [6], while a decreased monocyte-to-lymphocyte ratio (MLR) was found to significantly correlate with superior outcomes in patients receiving first-line therapy with PD-1 inhibitors plus chemotherapy [7]. In pancreatic carcinoma patients treated with PD-1 inhibitors, the predictive value of the pretreatment dNLR and LDH level for clinical outcome has also been discussed and validated [8]. In unresectable esophageal squamous cell carcinoma (ESCC), an MLR-based nomogram reliably predicted survival in patients treated with first-line therapy comprising an anti-PD-1 antibody plus systematic chemotherapy [9]. In NPC, the serum biomarkers EBV DNA and LDH were identified as two independent prognostic factors of survival in patients treated with PD-1 antibody-based immunochemotherapy [10]. Therefore, it is reasonable to generate a novel predictive model of PD-1 antibody efficacy based on these serum markers in a wide range of pan-cancer subtypes.

In contrast to gene expression profiling or immunohistochemistry of tumor tissue, serum markers from blood tests have advantages in terms of affordability, feasibility, non-invasiveness, reproducibility, and dynamic detection in clinical settings. Moreover, the standard detection of these serum markers, mostly derived from CBC and routine biochemistry tests, could be easily achieved in different centers and institutes. Up to one hundred parameters derived from CBC and routine biochemistry tests could be screened for their potential predictive value for PD-1 antibodies. Our previous study has demonstrated that dynamic changes in LDH levels and the serum ALT/AST ratio (LSR) significantly correlate with the efficacy of PD-1 antibodies in NPC patients. [11]

Machine learning, which encompasses multiple technologies for computationally simulating human intelligence, has achieved unprecedented success in diagnosing cancer, predicting cancer-related treatments and predicting patient survival. [12–16] In the study of developing blood index models through machine learning techniques, the utilization of circulating cytokine features in machine learning algorithms shows promise in predicting the efficacy of immune therapy in patients with non-small cell lung cancer, thus offering potential benefits for informing pre-treatment and early clinical decision-making processes. [17] However, machine learning methods have not yet been employed to establish predictive models for the efficacy of anti-PD-1 combination therapy in pan-cancer.

In the present study, we aimed to construct a pan-cancer predictive model for PD-1 antibody combination therapy. We employed multiple machine learning methods, including support vector machine (SVM), random forest (RF), adaptive boosting decision tree (AdaBoost), gradient boosting decision tree (GBDT), extreme gradient boosting decision tree (XGBoost) and artificial neural network (ANN) methods, to analyze the data derived from CBC and routine biochemistry tests of patients with NPC, ESCC and LC at baseline and after two cycles of PD-1 antibody combination therapy. Ultimately, the AdaBoost model for PD-1 antibody therapy prediction in pan-cancer patients was established and validated.

**Fig. 1:**
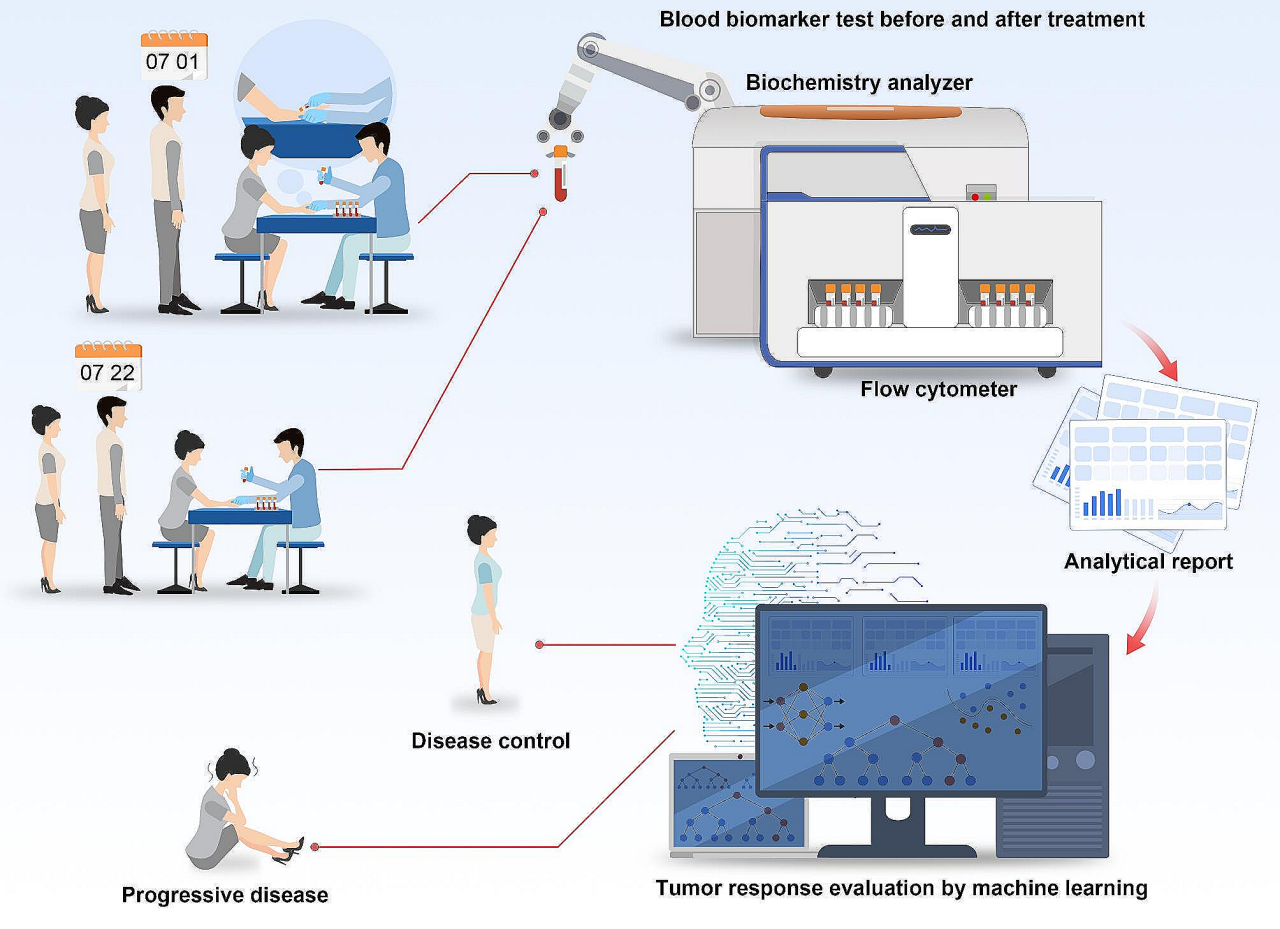
Machine learning-assisted prediction of cancer treatment response categories. Blood biomarker tests were also conducted before and after PD-1 antibody combination therapy. The tumor responses were diagnosed after 8–12 weeks of therapy. A biochemistry analyzer and flow cytometer were used to analyze the biomarkers. The indicators are implemented in machine learning models, and the tumor response is subsequently predicted to demonstrate the efficacy of therapy in patients

## Method

### Patients and study design

This retrospective study included 170 NPC patients, 110 esophageal cancer patients (ECs), and 151 lung cancer (LC) patients from Sun Yat-sen University Cancer Center who underwent PD-1 checkpoint inhibitor combination therapy. (Fig. 1) The inclusion criteria consisted of patients with NPC, ESCC or LC who received PD-1 therapy in combination with radiotherapy or/and chemotherapy or/and surgical treatment or/and targeted therapy from September 2018 to July 2022. The exclusion criteria consisted of a follow-up time of < 1 year, a lack of hematological examination before treatment and after the third week of treatment, a duration of PD-1 inhibitor administration of less than three months, and not undergoing imaging evaluation within 8–12 weeks. Imaging evaluations were carried out according to the Response Evaluation Criteria in Solid Tumors (RECIST) v1.1 to evaluate the effect of immunotherapy at 8–12 weeks and included progressive disease (PD) and non-PD (complete response (CR), partial response (PR), and stable disease (SD).

Basic clinical parameters, including age, sex, histological type, metastasis stage, and TNM classification, were collected.

### Laboratory examination

The complete blood analysis results were obtained using an automated XN-2000 hematology analyzer (Sysmex, Japan). Flow cytometry, impedance cytometry and optical cytometry were used to determine the hematological parameters of the Sysmex XN-2000 strain. The impedance method and hydrodynamic focusing method were used to count red blood cells (RBCs) and platelets. Fluorescent flow cytometry was used to determine the white blood cell (WBC) count in all the channels. Fluorescent flow cytometry was performed with scattered laser light (on the front and side). The Sysmex XN-2000 analyzer can be used to determine 28 basic diagnostic parameters and 16 optional diagnostic parameters, including RBC, WBC (percentage and absolute number of neutrophils, lymphocytes, eosinophils, basophils and monocytes), mean corpuscular volume (MCV), hematocrit (HCT), platelet (PLT), hemoglobin (HGB), mean corpuscular hemoglobin concentration (MCHC), and mean corpuscular hemoglobin (MCH).

Biochemical parameters were measured according to standard commercially available assays adapted to a Roche Cobas C702 Chemistry Analyzer (Roche Diagnostics, Japan) or Hitachi LABOSPECT 008 AS Chemistry Analyzer (Hitachi High-Tech Corporation, Japan) using automated procedures: glucose(GLU), urea, creatinine(CRE), uric acid(UA), total bile acid(TBA), triglycerides (TG), total cholesterol(CHO), aspartate aminotransferase (AST), alanine aminotransferase (ALT), alkaline phosphatase (ALP), gamma-glutamyl transferase (GGT), total proteins(TP), globulin(GLOB), albumin(ALB), carbon dioxide(CO2), calcium(Ca+), lactate dehydrogenase (LDH), total bilirubin(total bilirubin(TBA), Direct bilirubin(DBIL), cholinesterase (CHE), creatine kinase (CK), cystatin C(CYSC), high-density lipoprotein-C (HDL-C), low-density lipoprotein-C (LDL-C), apolipoprotein A1 (ApoA1), apolipoprotein B (ApoB), C-reaction protein(CRP), serum amyloid(SAA). A chemistry analyzer was used to conduct photometric assays on the absorbance changes of various analytes, and the quantitative results were calculated. The details are listed in Table S1 in the supplementary material.

The NLR, MLR, PLR, and systemic immune-inflammation index (SII) were calculated as the neutrophil count/lymphocyte count (NLR), monocyte count/lymphocyte count (MLR), platelet count/lymphocyte count (PLR), and NLR * platelet count, respectively.

### Machine learning methods for the prediction of cancer treatment response

To predict the response of cancer patients to PD-1 checkpoint inhibitor combinations, we employed commonly used machine learning methods, including principal component analysis (PCA), support vector machine (SVM) [18], random forest (RF) [19], adaptive boosting decision tree (AdaBoost) [20], gradient-boosting decision tree (GBDT) [21], extreme gradient boosting decision tree (XGBoost) [22] and artificial neural network (ANN) methods [23], to learn blood biomarker features. The dimension of the blood biomarker features was reduced to 2 in the PCA. We employed the ν-SVM method, which utilized a parameter ν to control the number of support vectors. After tuning the hyperparameters, ν was chosen to be 0.03, and the radial basis function was selected as the kernel function to maximize the prediction accuracy. For decision tree-based methods, multiple decision trees are employed to improve classification performance. The number of trees in the RF was set to 100, and the maximum depth of the trees was adjusted to 20. To evaluate the importance of blood biomarkers, base decision tree classifiers were used to calculate the feature importance in AdaBoost. The number of trees in AdaBoost was chosen to be 100, and the learning rate was 1. GBDT also employs 100 decision trees with a maximum depth of 3. For the XGB method, the tree number was adjusted to 60 with a maximum depth of 20. The ANN method employed 3 layers of neural networks, and the nodes were 64, 48 and 16 for the first, second and third hidden layers, respectively. We used the ReLU activation function for the first and second hidden layers. For the third output layer, the Softmax activation function was chosen to determine the probabilities for different class predictions. The optimization function of the MLP was the Adam function, and the learning rate was 0.0001. The loss function was chosen to be MSELoss.

### Response category prediction strategy

The blood biomarker levels of 431 patients were normalized to the range of [-1,1]. The numbers of patients with different treatment responses were 66 (PD), 256 (SD) and 109 (PR). The SD and PR patients formed the DC group. To train and test the machine learning models, the samples were randomly divided into training and testing datasets at a ratio of 8:2. In the training dataset, there were 345 patients, including 53 PD patients and 292 DC patients. Due to the imbalance between the numbers of PD patients and DC patients, the number of PD patients was increased from 53 to 292 with the synthetic minority oversampling technique (SMOTE) to avoid ignoring the features of PD during training [24]. In the testing dataset, there were 13 PD patients and 73 DC patients. The test results of the machine learning models are shown with receiver operating characteristic (ROC) curves. The scaled values of true positives, false positives, true negatives and false negatives are presented.

## Results

### Statistical analysis of blood biomarkers for cancer

The clinical characteristics of 170 NPC patients, 110 EC patients, and 151 LC patients who received PD-1 checkpoint inhibitor combination therapy are presented in Table 1.

**Table 1:** Demographics and clinical characteristics of patients

	NPC(*n* = 170)	EC(*n* = 110)	LC(*n* = 151)
	No. (%)	No. (%)	No. (%)
**Age, y**			
Median (range)	46 (19–78)	60 (42–78)	61 (30–90)
**Sex**			
Female	47 (27.65)	15 (13.64)	39 (25.83)
Male	123 (72.35)	95 (86.36)	112 (74.17)
**Histological type**			
Squamous cell carcinoma	170 (100)	99 (90)	14 (9.27)
Adenocarcinoma	0 (0)	5 (4.55)	127 (84.11)
Small cell carcinoma	0 (0)	4 (3.64)	2 (1.32)
Other	0 (0)	2 (1.82)	8 (5.3)
**Clinical stage**			
I-II	11 (6.47)	7 (6.36)	2 (1.32)
III	49 (28.82)	35 (31.82)	20 (13.25)
IV	110 (64.71)	68 (61.82)	129 (85.43)
**Metastasis stage**			
M0	82 (48.24)	71 (64.55)	23 (15.23)
M1	88 (51.76)	39 (35.45)	128 (84.77)
**The PD1 drug**			
Camrelizumab	21 (12.35)	3 (2.73)	4 (2.65)
Pembrolizumab	3 (1.76)	1 (0.91)	119 (78.81)
Toripalimab	124 (72.94)	81 (73.64)	1 (0.66)
Sintilimab	22 (12.94)	23 (20.91)	1 (0.66)
Nivolumab	0 (0)	2 (1.82)	24 (15.89)
Tislelizumab	0 (0)	0 (0)	2 (1.32)
**Outcomes**			
non-PD	23 (13.53)	17(15.45)	26 (17.22)
PD	147 (86.47)	93(84.55)	125 (82.78)

**Fig. 2:**
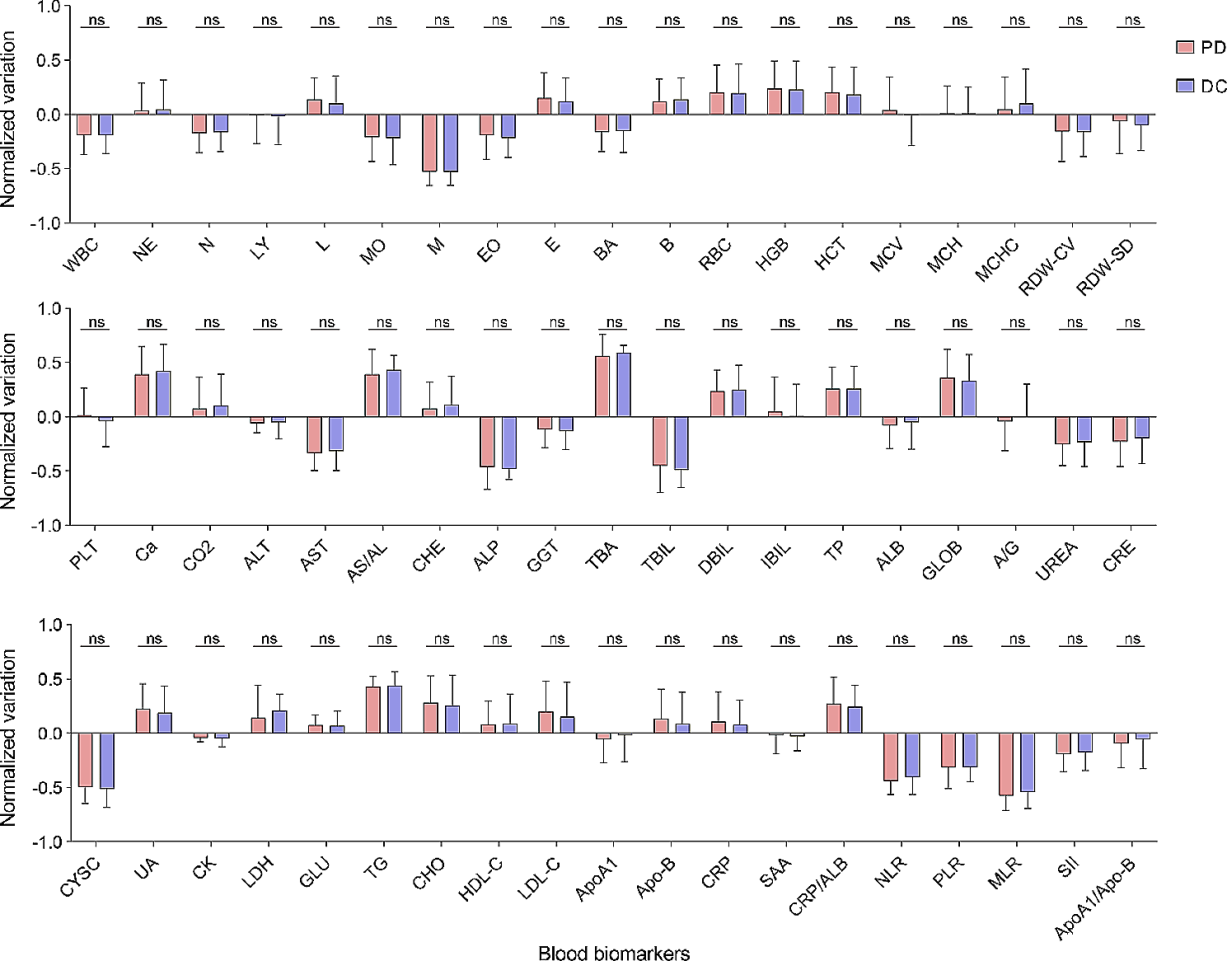
Statistical analysis of blood biomarkers in the testing dataset. * denotes *p* < 0.05, ** denotes *p* < 0.01, *** denotes *p* < 0.001, ns denotes not significant

We conducted an analysis to examine the difference in the changes in the levels of 57 blood biomarkers before treatment and after three weeks of PD-1 inhibitor treatment (∆W3) between the PD and DC groups. (Fig. 2) None of the indicators were significantly different between the PD and DC groups.

### Heatmap and PCA of blood biomarkers for cancer

**Fig. 3:**
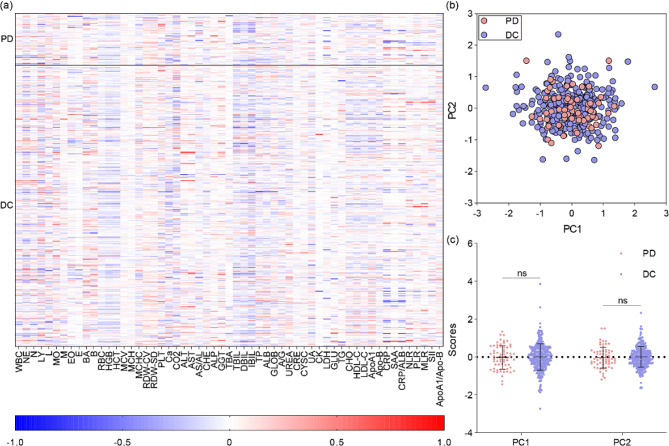
Traditional analysis of blood biomarkers for predicting response category. (**a**) Heatmap of biomarkers of PD and DC patients. (**b**) PCA of PD and DC patient blood biomarkers with dimension reduction to 2. (**c**) Statistical analysis of PCs from PCA. * denotes *p* < 0.05, ** denotes *p* < 0.01, *** denotes *p* < 0.001, ns denotes not significant

To further analyze the blood biomarkers for tumor response prediction, we examined the normalized variations in biomarkers after treatment via traditional analyses, including heatmaps and PCA (Fig. 3). There was no significant difference between PD and DC (Fig. 3a). For example, several blood biomarkers, including RBC, HGB, HCT, total bilirubin (TBIL), direct bilirubin (DBIL) and indirect bilirubin (IBIL), from both the PD and DC groups generally showed a decreasing trend. Furthermore, BA, B, RDW-CV, RDW-SD, ALT, AST, ALP and GGT in both groups generally increased after treatment. For some blood biomarkers, including EO, E, TBA, LDH and GLU, in the PD and DC groups, most of the patients exhibited little variation, while some patients exhibited prominent increasing or decreasing values. Biomarkers, including MCV, MCH, CRE, CYSC and CK, have little variation between PD patients and DC patients. Moreover, most of the blood biomarkers, including WBC, NE, N, LY, L, MO, M, MCHC, PLT, Ca, CO2, AS/AL, CHE, TP, ALB, GLOB, A/G, UREA, UA, TG, CHO, HDL-C, LDL-C, ApoA1, Apo-B, CRP, SAA, CRP/ALB, NLR, PLR, MLR, SII and ApoA1/Apo-B, exhibited inconsistent variations between the PD and DC groups. Similarly, according to the PCA results, the PD and DC patients could not be differentiated from the overlapping samples after dimension reduction (Fig. 3b). The student’s t test of principal components between PD and DC groups also showed no difference (*p* = 0.83 for PC1 and *p* = 0.65 for PC2). In summary, neither the heatmap nor the PCA could predict PD patients according to comparisons of the PD and DC groups.

### Prediction of Pancancer treatment responses using machine learning methods

**Fig. 4:**
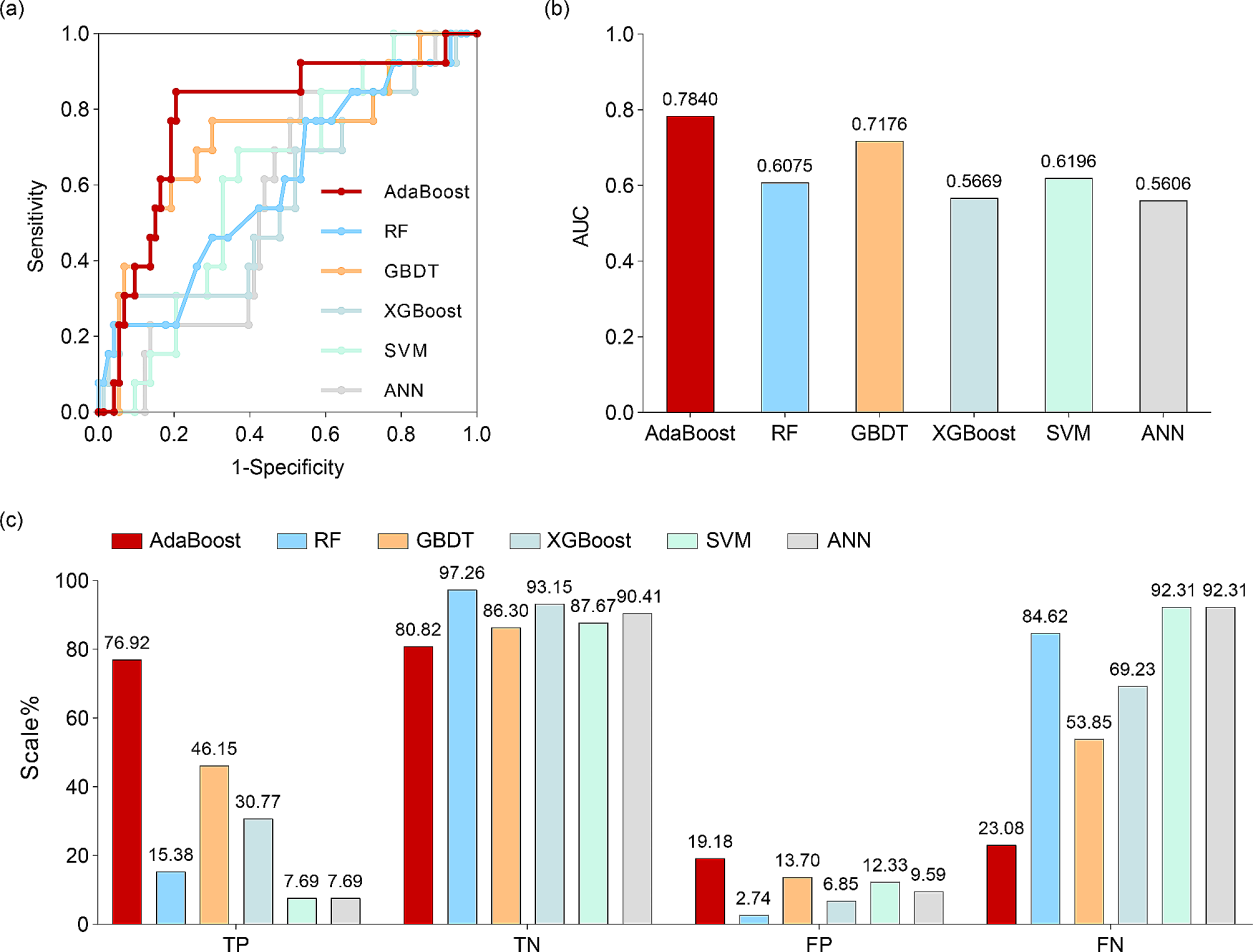
Prediction via machine learning methods. (**a**) ROC curves of the applied machine learning methods for PD prediction. (**b**) AUC values of the machine learning methods. (**c**) True positives (TPs), true negatives (TNs), false positives (FPs) and false negatives (FNs) predicted by the machine learning methods

To predict treatment responses in cancer patients, we developed and trained 6 machine learning models (Fig. 4). The ROC curves of the predictions show that the AdaBoost classifier achieves the best performance among these methods. The sensitivity reached 0.85 when the 1-specificity was 0.21 (Fig. 4a). The GBDT method also achieved better classification than did the other methods. Both AdaBoost and GBDT achieved an AUC ≥ 0.70, which is generally accepted as clinically useful (Fig. 4b). However, other methods have AUCs lower than 0.62. To evaluate the prediction performance for PD and DC, we further presented the predicted numbers of true positives, true negatives, false positives and false negatives (Fig. 4c). All the algorithms can excellently predict true negatives (DCs) with an accuracy greater than 80%, and few patients are classified as false positives. The AdaBoost algorithm predicts the highest number of true positives in PD patients with an accuracy of 76.92%, and it can also predict DC patients (true negatives) with an accuracy of 80.82%. Although GBDT achieved a true negative prediction accuracy of 97.26%, it failed to correctly predict most of the PD patients and had an accuracy of 46.15%. With respect to the RF, XGBoost, SVM and ANN methods, more than 69% of the PD patients were falsely predicted to be negative. In summary, the results demonstrate that the AdaBoost method has the best performance when compared to other machine learning methods and predicts PD with clinical accuracy.

### Variable importance

**Fig. 5:**
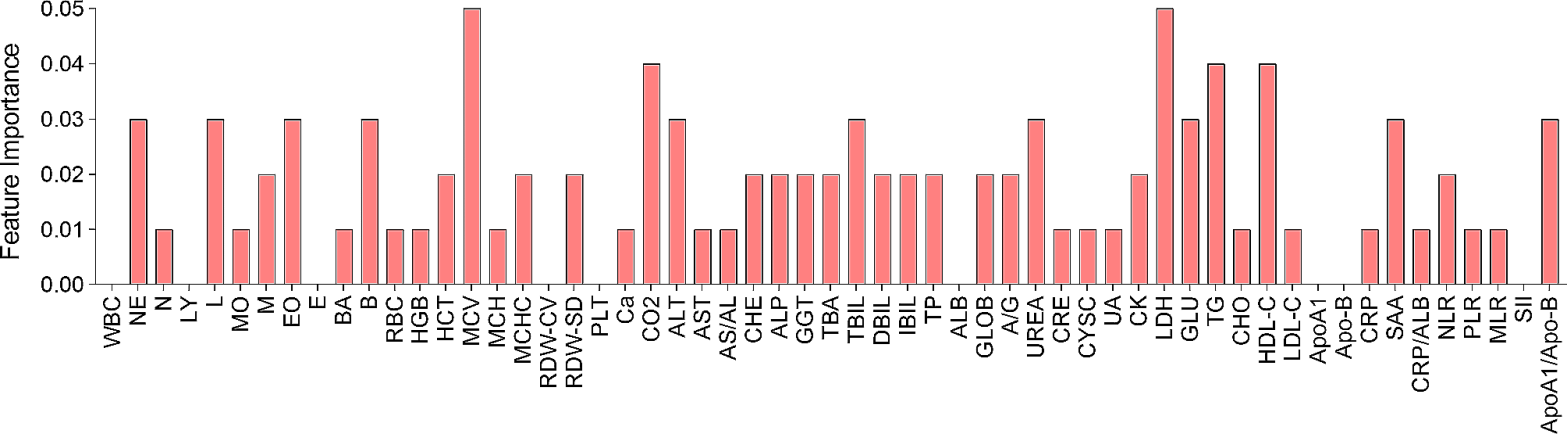
Normalized feature importance scores of AdaBoost analysis

The feature importance analysis of AdaBoost is shown in Fig. 5. The result demonstrates that the normalized feature importance scores are all lower than 0.05. MCV and LDH have the highest scores, implying that they contribute most to the model accuracy.

## Discussion

PD-1 inhibitors are widely utilized in cancer therapy, but the main challenges in developing successful drugs for immune checkpoint blockade are the selection of patient subgroups that would benefit most from these agents and the avoidance of ineffective treatments and potential side effects related to autoimmune effects resulting from blocking the PD-1/PD-L1 pathway [25, 26]. Nevertheless, the current indicators have demonstrated limited efficacy, and some necessitate the acquisition of tissue samples, incurring high costs [27]. For example, the limitations of PD-L1 immunohistochemical expression include intratumor heterogeneity, the lack of standardized scoring criteria, and the potential for distinguishing between biopsy samples and metastatic sites. These limitations highlight several key challenges in accurately assessing PD-L1 expression [28, 29]. Similarly, the tumor microenvironment (TME) is recognized as a critical component influencing tumor progression and the immune response. However, the assessment of the TME is limited by the lack of standardized methods and comprehensive characterization techniques [30]. Given the limited response rates of patients with advanced tumors to immune checkpoint inhibitors (which range between 20% and 50%) [31], it is imperative to explore and identify noninvasive markers that can be used to effectively evaluate the efficacy of immunotherapy.

Recently, several studies have proposed the use of serum-based indicators, such as the neutrophil-to-lymphocyte ratio (NLR), to predict treatment response [32]. Alessi et al. reported that a high baseline dNLR and an increase in the dNLR between cycle 1 and cycle 2 in NSCLC patients treated with pembrolizumab monotherapy prior to radiological assessment are associated with worse clinical outcomes [33]. In our previous investigation, we devised a model employing lactate dehydrogenase (LDH) and the alanine transaminase/aspartate transaminase (ALT/AST) ratio for predicting the efficacy of PD-1 inhibitors in patients with NPC [11]. In this study, we further assessed the predictive value of serum-based indicators by applying machine learning techniques to a broader range of cancer patients receiving PD-1 inhibitor treatment. First, we examined the changes in routine hematologic and biochemical parameters before treatment and at the third week in patients receiving PD-1 therapy. Among the 57 indicators, none of them exhibited a significant difference between the DC and PR groups. Traditional statistical analysis methods cannot help demonstrate their ability to predict therapeutic outcomes. Subsequently, we employed multiple machine learning methods to explore the predictive value of dynamic serum biomarkers.

Various machine learning models have been employed in clinical and translational oncology, leveraging a blend of clinical data to improve patient diagnosis, prognosis, and treatment selection [34–36]. Therefore, we developed and trained 6 machine learning models including SVM, RF, AdaBoost, GBDT, XGBoost and ANN methods, to analyze the data derived from CBC and routine biochemistry tests of patients with NPC, ESCC and LC at baseline and after two cycles of PD-1 antibody combination therapy to predict the efficacy of PD-1 combination therapy at 8–12 weeks. With our predictive model, we found that both the AdaBoost classifier and GBDT demonstrated high levels of prediction efficiency, with AUC values exceeding 0.7. This study revealed that the AdaBoost classifier (AUC = 0.784; sensitivity = 0.85; specificity = 0.79) could accurately predict the effectiveness of PD-1 combination therapy. For the first time, our research employed various machine learning techniques to assess the efficacy of PD-1 therapy in patients with pan-cancer. The performance of the AdaBoost classifier, with an AUC of 0.784, surpassed that of our previously developed models based on alanine transaminase (ALT)/aspartate transaminase (AST) and lactate dehydrogenase (LDH) levels, which yielded AUC values of 0.737 in the training cohort and 0.723 in the validation cohort [11]. To streamline the model’s parameters, numerous studies employ feature importance analysis to identify significant feature parameters and develop revised models. In the present study, we similarly conducted an analysis of feature importance within the model; however, all parameters yielded scores below 0.05, thus precluding the need for further simplification of the model.

Our machine learning-based approach demonstrated promising accuracy in predicting the efficacy of PD-1 therapy at 8–12 weeks. These findings have significant implications for personalized medicine and the optimization of treatment strategies for patients receiving PD-1 therapy. By identifying patients who are likely to respond positively to PD-1 therapy, we can optimize treatment plans and avoid unnecessary side effects and costs for nonresponders. This approach has the advantage of being noninvasive and easily applicable in clinical settings.

Although, studies have been conducted on radiomic data using machine learning to predict the efficacy of anti-PD-1 antibodies-based combinational treatment in advanced breast cancer, demonstrating good agreement with the actual clinical immunotherapy response status. (AUC of 0.994 in the training cohort, and 0.920 in the validation cohort) [3] Zhao et al. has not conducted an assessment of the blood indicators, thus hindering a comprehensive understanding of the efficacy between blood and radiomic model. Nevertheless, the simplicity and affordability of utilizing blood indicators present clear advantages for imaging purposes. Especially hematological testing is more suitable for repeated testing in a short period of time, with a shorter warning interval.

While this study marks the initial exploration into forecasting the effectiveness of PD-1 combination therapy across diverse cancer types, it is important to acknowledge its limitations. First, this study is limited by a small sample size and the lack of external multicenter datasets and representation of other cancer types. Future research should aim to validate the predictive model in diverse cancer types and across multiple research centers. Second, additional non-invasive prediction models, such as imaging parameters models, have shown promising results in enhancing the predictive accuracy of PD-1. The potential synergistic effects of combining blood and imaging omics for enhanced predictive capabilities warrant further investigation in future studies. Third, our study focused on a specific time frame (8–12 weeks) and may not capture long-term treatment outcomes.

## Conclusions

In conclusion, our research demonstrated the potential of machine learning to predict the efficacy of PD-1 therapy based on changes in routine blood cell and biochemical parameters. This innovative approach holds the potential to enhance the monitoring of patient treatment plans and optimize treatment efficiency by accurately predicting the efficacy of PD-1 combination therapy.

### Electronic supplementary material

Below is the link to the electronic supplementary material.

Supplementary Material 1

Supplementary Material 2

## Data Availability

No datasets were generated or analysed during the current study.
