# Supplementary material for: Machine learning models for predicting of PD-1 treatment efficacy in Pan-cancer patients based on routine hematologic and biochemical parameters
Source: Cancer Cell Int. 2024 Jul 21;24:258. doi: 10.1186/s12935-024-03439-6 (PMC11265142; doi:10.1186/s12935-024-03439-6)
Supplement: Supplementary file 1 — Supplementary Material 1 [file 12935_2024_3439_MOESM1_ESM.docx]

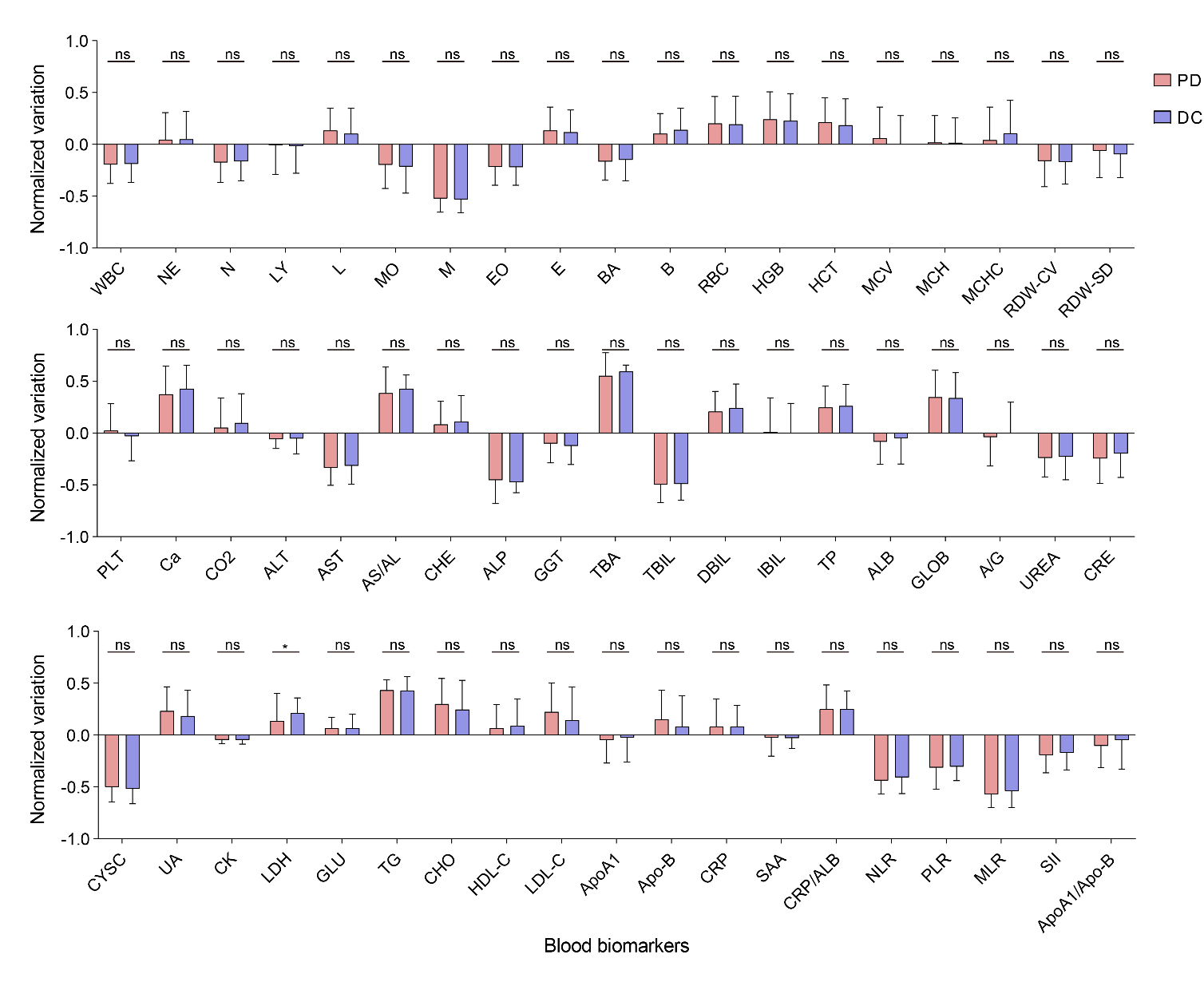


**Fig. S1**. Statistical analysis of blood biomarkers of training dataset. * denotes p<0.05, ** denotes p<0.01, *** denotes p<0.001, ns denotes not significant.


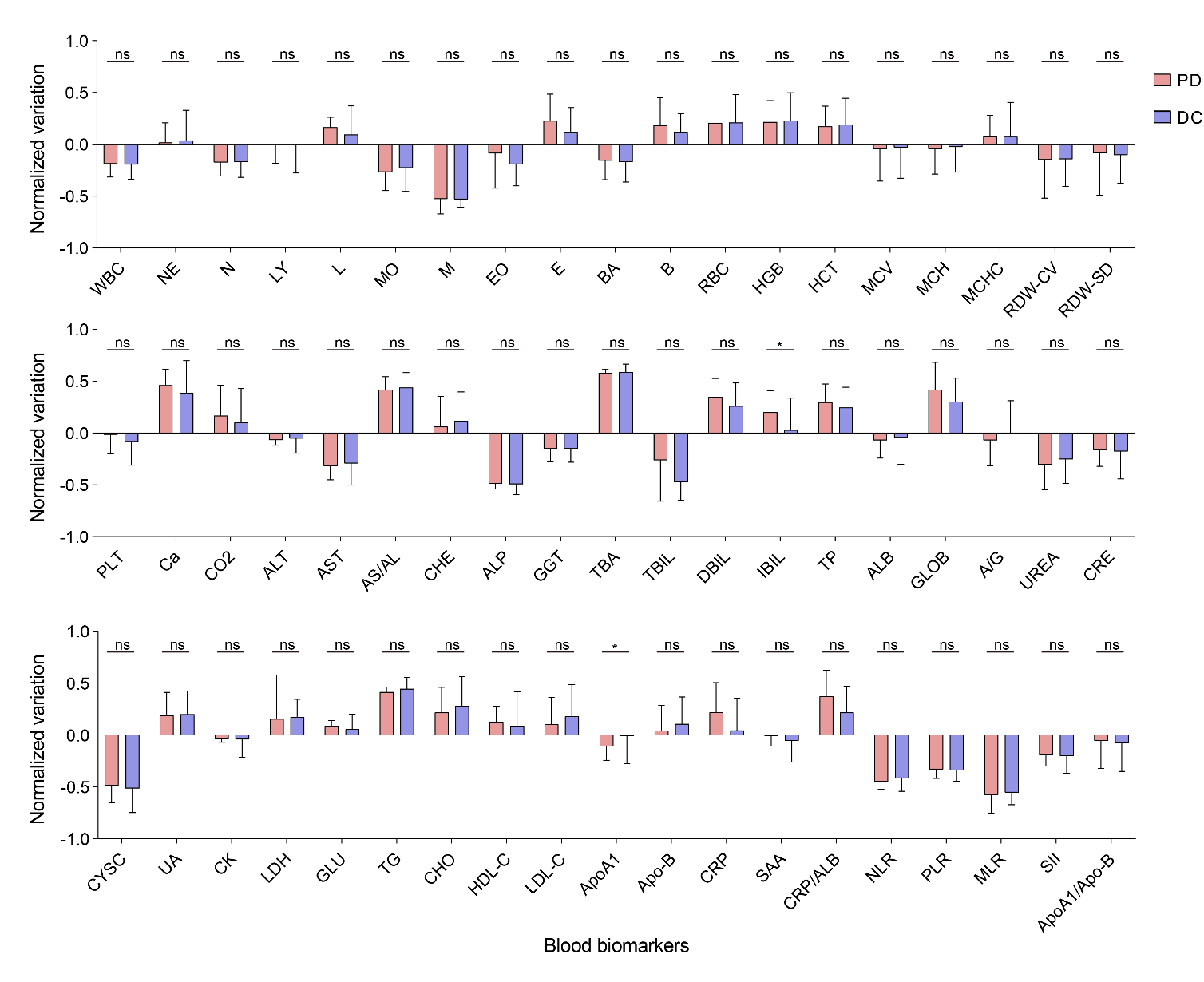


**Figure S2**. Statistical analysis of blood biomarkers of testing dataset. * denotes p<0.05, ** denotes p<0.01, *** denotes p<0.001, ns denotes not significant.
