# Supplementary material for: Machine learning models for predicting of PD-1 treatment efficacy in Pan-cancer patients based on routine hematologic and biochemical parameters
Source: Cancer Cell Int. 2024 Jul 21;24:258. doi: 10.1186/s12935-024-03439-6 (PMC11265142; doi:10.1186/s12935-024-03439-6)
Supplement: Supplementary file 2 — Supplementary Material 2 [file 12935_2024_3439_MOESM2_ESM.docx]

Supplementary Table.S1: Detection methods and platforms for hematologic and biochemical parameters

| [Test](C:/Program%20Files%20(x86)/Youdao/Dict/8.9.6.0/resultui/html/index.html#/javascript:;) [item](C:/Program%20Files%20(x86)/Youdao/Dict/8.9.6.0/resultui/html/index.html#/javascript:;) | [Test](C:/Program%20Files%20(x86)/Youdao/Dict/8.9.6.0/resultui/html/index.html#/javascript:;) [method](C:/Program%20Files%20(x86)/Youdao/Dict/8.9.6.0/resultui/html/index.html#/javascript:;) | [Instrument](C:/Program%20Files%20(x86)/Youdao/Dict/8.9.6.0/resultui/html/index.html#/javascript:;) |
| --- | --- | --- |
| Ca2+  CO2  CK  CHE  BUN  SAA  CRP  ALT  CYSC  HDL  LDL  TBIL  DBIL  GGT  TP  ApoA1  ApoB  AST  ALP  LDH  TBA  TG  CHO  GLU  UA  CRE  ALB  WBC  WBC [differential](C:/Users/47963/AppData/Local/youdao/dict/Application/8.9.9.0/resultui/html/index.html#/javascript:;) count  RBC&PLT  HGB  NLR  PLR  MLR  SII  AST/ALT  IBIL  GLB  A/G  NE  LY  MO  EO  BA  HCT  MCV  MCH  MCHC  RDW-CV  RDW-SD | Colorimetry  Methyl alkyl dimethyl phenol blue method  Colorimetry  PEPC enzymatic method  Ultraviolet spectrophotometry  Continuous monitoring assay  Colorimetry  Methyl thiophenoyl thiocholine method  Continuous monitoring assay/Enzyme assay  Latex Enhanced Immunoturbidimetric method  Latex Immunoturbidimetric method  Continuous monitoring assay(IFCC)  Latex immunological turbidimetry  Homogeneous enzyme colorimetry  Homogeneous enzyme colorimetry  Diazo method/Vanadate oxidation  Diazo method/Vanadate oxidation  Enzyme colorimetry/Rate assay  Colorimetry/[Biuret](C:/Users/47963/AppData/Local/youdao/dict/Application/8.9.9.0/resultui/html/index.html#/javascript:;) [method](C:/Users/47963/AppData/Local/youdao/dict/Application/8.9.9.0/resultui/html/index.html#/javascript:;)  [Immunoturbidimetry](C:/Users/47963/AppData/Local/youdao/dict/Application/8.9.9.0/resultui/html/index.html#/javascript:;)  [Immunoturbidimetry](C:/Users/47963/AppData/Local/youdao/dict/Application/8.9.9.0/resultui/html/index.html#/javascript:;)  Colorimetry/Rate assay  Colorimetry/Rate assay  Colorimetry/Rate assay  Enzyme Cycling method  Enzyme colorimetry  Enzyme colorimetry  Hexokinase method  Enzyme assay  Enzyme assay  Colorimetry  Flow cytometry  Flow cytometry  Sheath flow or Dc impedance method  SLS- Hemoglobin test  Neutrophil-to-lymphocyte ratio  Platelet-to-lymphocyte ratio  Monocyte-to-lymphocyte ratio  PLT multiplied by NE then divided by LY  AST-to-ALT ratio  TBIL minus DBIL  TP minus ALB  ALB-to-GLB ratio  N-to-WBC ratio  L-to-WBC ratio  M-to-WBC ratio  E-to-WBC ratio  B-to-WBC ratio  Total red blood cell volume / Total blood volume  10*HCT-to-RBC ratio  10E12*HGB-to-RBC ratio  MCH-to-MCV ratio  calculated by RBC histogram  calculated by RBC histogram | ROCHEc702  [Hitachi](C:/Program%20Files%20(x86)/Youdao/Dict/8.9.6.0/resultui/html/index.html#/javascript:;)008  ROCHEc702  [Hitachi](C:/Program%20Files%20(x86)/Youdao/Dict/8.9.6.0/resultui/html/index.html#/javascript:;)008  ROCHEc702  [Hitachi](C:/Program%20Files%20(x86)/Youdao/Dict/8.9.6.0/resultui/html/index.html#/javascript:;)008  ROCHEc702  [Hitachi](C:/Program%20Files%20(x86)/Youdao/Dict/8.9.6.0/resultui/html/index.html#/javascript:;)008  ROCHEc702/[Hitachi](C:/Program%20Files%20(x86)/Youdao/Dict/8.9.6.0/resultui/html/index.html#/javascript:;)008  ROCHEc702/[Hitachi](C:/Program%20Files%20(x86)/Youdao/Dict/8.9.6.0/resultui/html/index.html#/javascript:;)008  ROCHEc702/[Hitachi](C:/Program%20Files%20(x86)/Youdao/Dict/8.9.6.0/resultui/html/index.html#/javascript:;)008  ROCHEc702/[Hitachi](C:/Program%20Files%20(x86)/Youdao/Dict/8.9.6.0/resultui/html/index.html#/javascript:;)008  ROCHEc702/[Hitachi](C:/Program%20Files%20(x86)/Youdao/Dict/8.9.6.0/resultui/html/index.html#/javascript:;)008  ROCHEc702/[Hitachi](C:/Program%20Files%20(x86)/Youdao/Dict/8.9.6.0/resultui/html/index.html#/javascript:;)008  ROCHEc702/[Hitachi](C:/Program%20Files%20(x86)/Youdao/Dict/8.9.6.0/resultui/html/index.html#/javascript:;)008  ROCHEc702/[Hitachi](C:/Program%20Files%20(x86)/Youdao/Dict/8.9.6.0/resultui/html/index.html#/javascript:;)008  ROCHEc702/[Hitachi](C:/Program%20Files%20(x86)/Youdao/Dict/8.9.6.0/resultui/html/index.html#/javascript:;)008  ROCHEc702/[Hitachi](C:/Program%20Files%20(x86)/Youdao/Dict/8.9.6.0/resultui/html/index.html#/javascript:;)008  ROCHEc702/[Hitachi](C:/Program%20Files%20(x86)/Youdao/Dict/8.9.6.0/resultui/html/index.html#/javascript:;)008  ROCHEc702/[Hitachi](C:/Program%20Files%20(x86)/Youdao/Dict/8.9.6.0/resultui/html/index.html#/javascript:;)008  ROCHEc702/[Hitachi](C:/Program%20Files%20(x86)/Youdao/Dict/8.9.6.0/resultui/html/index.html#/javascript:;)008  ROCHEc702/[Hitachi](C:/Program%20Files%20(x86)/Youdao/Dict/8.9.6.0/resultui/html/index.html#/javascript:;)008  ROCHEc702/[Hitachi](C:/Program%20Files%20(x86)/Youdao/Dict/8.9.6.0/resultui/html/index.html#/javascript:;)008  ROCHEc702/[Hitachi](C:/Program%20Files%20(x86)/Youdao/Dict/8.9.6.0/resultui/html/index.html#/javascript:;)008  ROCHEc702/[Hitachi](C:/Program%20Files%20(x86)/Youdao/Dict/8.9.6.0/resultui/html/index.html#/javascript:;)008  ROCHEc702/[Hitachi](C:/Program%20Files%20(x86)/Youdao/Dict/8.9.6.0/resultui/html/index.html#/javascript:;)008  ROCHEc702/[Hitachi](C:/Program%20Files%20(x86)/Youdao/Dict/8.9.6.0/resultui/html/index.html#/javascript:;)008  ROCHEc702/[Hitachi](C:/Program%20Files%20(x86)/Youdao/Dict/8.9.6.0/resultui/html/index.html#/javascript:;)008  ROCHEc702/[Hitachi](C:/Program%20Files%20(x86)/Youdao/Dict/8.9.6.0/resultui/html/index.html#/javascript:;)008  ROCHEc702/[Hitachi](C:/Program%20Files%20(x86)/Youdao/Dict/8.9.6.0/resultui/html/index.html#/javascript:;)008  ROCHEc702/[Hitachi](C:/Program%20Files%20(x86)/Youdao/Dict/8.9.6.0/resultui/html/index.html#/javascript:;)008SYSMEX XN9000/2000  SYSMEX XN9000/2000  SYSMEX XN9000/2000  SYSMEX XN9000/2000  NA  NA  NA  NA  NA  NA  NA  NA  NA  NA  NA  NA  NA  NA  NA  NA  NA  NA  NA |
| CRP/ALB | CRP-to-ALB ratio | NA |
| ApoA1/ApoB | ApoA1-to-ApoB ratio | NA |

WBC differential Count contains N (neutrophil), L (lymphocyte), M (monocyte), E (eosinophil), B (basophils)
